# Knowledge of, Perception of, and Attitude towards Uterine Fibroids among Women with Fibroids in Lagos, Nigeria

**DOI:** 10.1155/2014/809536

**Published:** 2014-03-13

**Authors:** M. A. Adegbesan-Omilabu, K. S. Okunade, A. Gbadegesin

**Affiliations:** ^1^Department of Obstetrics & Gynaecology, Lagos University Teaching Hospital (LUTH), Lagos, Nigeria; ^2^Department of Obstetrics & Gynaecology, Lagos State University Teaching Hospital (LASUTH), Lagos, Nigeria

## Abstract

*Objectives*. The study was to assess the level of knowledge of, perception of, and attitude towards uterine fibroids among women diagnosed with the condition. *Methods*. It is a cross-sectional descriptive study carried out among women diagnosed as having uterine fibroids in two gynaecological clinics in Lagos, Nigeria. Eligible women were recruited and a structured interviewer-administered questionnaire was used to collect the required information. Statistical analysis of data was done using EPI Info 2008. *Results*. Knowledge of fibroids was reported in 98.6% of the respondents and the information on uterine fibroids was obtained from radio, parents/relatives, health workers, and television in 29%, 27.3%, 18.7%, and 18.3%, respectively, by the respondents. Most of the women believed that being black, being nulliparous, or having positive family history predisposes women to having uterine fibroids. Up to 69.0% of the respondents believed that fibroid is a spiritual problem and many thought it requires spiritual healing. Fear of complications of surgery keeps most sufferers away from the hospital until fibroids become advanced or associated with complications. *Conclusion*. Awareness of uterine fibroids is high, but correct knowledge on aetiology and proper treatment is low. Intensive enlightenment of the populace using the mass media by trained personnel is recommended.

## 1. Introduction

Uterine leiomyoma commonly or uterine fibroid is the most common of all pelvic tumours in women. It is composed essentially of muscle tissue although there is a variable amount of fibrous connective tissue as well [1].

The incidence of uterine fibroid depends on age and race [1]. It is quite high in Nigerian women with over 80% of those above 25 years of age having fibroids if only of the size of a seedling [2]. The vast majority of these fibroids are not symptomatic [3, 4]. Fibroids are more common in Negroes, 3–9 times more than in Caucasians [5]. They occur after menarche and the majority would undergo atrophy at menopause [5].

The precise aetiology of uterine fibroids is still unknown, but genetic determinants in addition to hormonal factors-oestrogen, growth hormone and epidermal growth factor, play a synergistic or facilitative role in their growth [6–10]. The predisposing factors for clinically significant fibroids are nulliparity, obesity, and a positive family history [8]. The combined oral contraceptive pill is said to be protective of the development of uterine fibroid in users [11].

The clinical symptoms and signs of uterine fibroid are variable with less than 50% being asymptomatic, one-third having abnormal uterine bleeding, and another one-third having pelvic pain usually acute, sequel to degenerative changes, torsion haemorrhage, or infection [12, 13]. Other clinical features include abdominal swelling, pelvic pressure, urinary frequency, compressive bowel symptoms, and subfertility. In Lagos and also in other parts of Nigeria, women with uterine fibroids present late to the hospital with large tumours (uterus greater than 20-week size gestation) which cause increased morbidity and mortality [2].

Diagnosis of these tumours may be clinically elicited from the history and examination or incidentally during abdominal palpation in pregnancy. Ultrasonography, hysterosalpingography, laparoscopy, and laparotomy are other diagnostic procedures for uterine fibroids. In addition, magnetic resonance imaging, computerized tomography, and endoscopic diagnostic methods can also be used [1].

The treatment options for uterine leiomyoma include medication, surgery, myolysis, and uterine artery embolization [14]. Surgery for removal of fibroids can be by either myomectomy or hysterectomy depending on the age and parity of the women and this could be performed by the abdominal or vaginal route. Recently, there is an increasing trend for minimal access surgery (endoscopic surgery) for treatment of uterine fibroid in developed countries [15]. Palliative treatment is also advocated to temporarily control abnormal uterine bleeding (menorrhagia) and this includes administration of danazol or norethisterone acetate, oestrogen-progesterone preparation such as in combined oral contraception, and gonadotrophin releasing hormone (GnRH) agonist [1, 15]. Uterine fibroid could also be treated by embolization [12].

The aim of this study therefore was to assess the knowledge, perception, and attitude of women who have uterine fibroids in Lagos regarding the condition and to determine the factors responsible for late presentation among these women. It is thus hoped that the result of this study would help to increase awareness and to bring in intervention to reduce late presentation by the affected women.

## 2. Materials and Methods

The study was a cross-sectional descriptive study carried out at the gynaecological outpatients clinics of two teaching hospitals in Lagos, Nigeria, with eligible participants recruited by consecutive sampling method.

Three hundred (300) women were enrolled into the study based on the minimum sample size calculated using the statistical formula by Fisher [16]. These women were consenting patients who had sonographically diagnosed uterine fibroids.

A structured and pretested interviewer-administered questionnaire containing both open and closed ended questions was administered to each consenting respondent. Information on sociodemographic indices of the respondents and their knowledge of, perception of, and attitude toward uterine fibroids was collected. Quantitative data was entered in computer and analysis done using Epi Info 2008 version 3.5.1 statistical software. Descriptive statistics were computed for all relevant data.

Ethical approval was obtained from the two hospitals' Health Research and Ethics Committee prior to the commencement of the study and written consent obtained from each participant before involvement in the study.

## 3. Results

Table 1 showed the demographic characteristics of the respondents. The age of the respondents ranged from 20 to 49 years with median age of 30.6 years. The majority of the respondents were within the age of 30–34 (28.0%) and 35–39 years (29.3%), respectively.

About two-thirds (66.7%) of the respondents were married and only 1.0% were widowed, respectively. The majority (52.7%) of the respondents in the study group were nulliparous while only 2.3% had a parity of more than 5. The occupational status of the respondents also varied with 88 (29.3%) unemployed while the rest were involved in one form of profession or the other (Table 1).

Table 2 showed that a significant proportion of the respondents presented late to the hospital for management (86.7%) while 13.3% presented early for treatment. Most of these women presented with abdominal mass (88.0%) while only a few presented with irregular bleeding (13.3%) and urinary frequency (10.0%), respectively. The majority of the respondents (88.7%) presented with uterine size corresponding to pregnancy size of 20 weeks and above while only 11.3% presented with fibroid size corresponding to less than 20-week size of gestation.

In Table 3, a vast majority (98.6%) of the respondents have heard about fibroids while only 1.4% had never heard of it. About one-third heard about fibroid from the radio, 27.3% from parent/relative, and 18.7% from the hospital, while 18.3% and 6.7% heard about it from television and newspapers, respectively. Table 3 also revealed that more than one-third of the women believed that fibroids were only seen in women who had never had a child and of the black race, respectively. Only 26.0% thought fibroids run in families.

In Table 4, the majority of the women (67.0%) perceived fibroids as a spiritual problem; 52.7% believed that fibroids affect child bearing while 32.3% perceived fibroids as a disease of the infertile women. Over 83% of the women expressed their fear of surgical complication as the reason for their late presentation to the hospital while about two-thirds (67.0%) of the women believed that treatment is best sought in the spiritual home (churches, mosques, and massage parlours). Almost one-third of the respondents managed their fibroids with local herbs while 29.7% of them use pharmacological agent (medical treatment) with drugs in treating the fibroids.

Table 5 showed the various drugs that the patients used before presentation. About 68.3% of these women had used drugs that contain phytoestrogens for treatment of uterine fibroids and various other conditions such as infertility and menstrual regulation prior to their presentation.

## 4. Discussion

In this study, the knowledge, perception, and attitude of Lagos women with uterine fibroids regarding the condition were reviewed. Apart from representing women in the reproductive age range, they also represented the majority of gynaecological patients. The study population was predominantly nulliparous [1], but it was observed that multiparous women were also affected. Similar observations had been made by some other authors [2, 5].

It was also observed in our study that the majority of the women were nulliparous and married which could actually explain the misconception about fibroids and infertility. Fibroids are an infrequent primary cause of infertility and have been reported as the sole cause in only a small proportion of infertile patients [6]. Most women with uterine fibroids have uncomplicated pregnancies and deliveries [6]. The risk of pregnancy complication/loss is influenced by both fibroids location and size [6].

The study showed high level of awareness of fibroid among the respondents (98.6%), although there is a high level of misconception about the disease. This may be a reflection of numerous advertisements and publicity generated by the traditional medical practitioners on both electronic and print media. Most of the respondents (33.3%) were aware that fibroids are commonly seen in nulliparous, obese, and black women. These are well-documented predisposing factors [5].

About one-fourth (26%) of these women were also aware that fibroids run in the family as documented by most authors [5, 8, 10, 17, 18]; perhaps this may be because these women have relatives that also have uterine fibroids.

Most of the women (67.0%) perceived fibroid as a spiritual problem and hence sought treatment from the spiritual homes and invariably presented late to the hospital. The fear of complication of surgery for fibroids made many seek alternative means of treatment. It is not unlikely that this fear of surgery was borne out of misinformation that they might have obtained from the mass media, although surgery for uterine fibroid is not without complications [19, 20].

A vast majority of the respondents, for many reasons (predominantly infertility), used certain pharmacological agents that were often oestrogen containing or local herbs that have been shown to contain phytoestrogen compounds. Many of the respondents have used most of these herbal products either singly or in combination with some orthodox drugs such as bromocriptine, cod liver oil, or clomiphene citrate at one point or the other in the past.

Clomiphene is a nonsteroidal agent with both oestrogenic and nonoestrogenic properties [21] that has been widely used for induction of ovulation in cases of anovulatory infertility since 1962 [22]. It acts by stimulating the release of follicle stimulating hormone (FSH) by blocking oestradiol secretion from the ovaries resulting in multiple follicular developments. This consequently leads to a rise in the secretion of endogenous oestrogen by ovaries. High level of endogenous oestrogen has been cited as one of the possible predisposing factors for uterine fibroid, as well as accelerating the growth of fibroids [6]. It is therefore not surprising that these women presented with very large fibroids. This is also seen in the large number of respondents who took phytoestrogen containing herbal medications prior to seeking treatment. Other herbal drugs such as ashwagandha [23] and Guizhi Fuling Formula [24] which are very revered herbs of the Asian Ayurvedic system of medicine have also been mentioned in the treatment of uterine fibroids among Asian women; however large scale clinical studies are still needed to prove the clinical efficacy and safety of these herbal preparations [24, 25], and other related herbs which were examined in this study.

## 5. Limitations to the Study

The study is hospital based and the findings may not be representative of the general women population. There was also selection bias in the enrolment of participants due to the incessant industrial actions in the health sector which further limits the generalizability of the study to the whole population.

## 6. Conclusion

The study has shown that there is generally poor knowledge about uterine fibroids. There are also a vast majority of women taking over-the-counter drugs in Nigeria that are likely to enhance the growth of fibroids. It is therefore recommended that intensive enlightenment about the aetiology and modality of treatment of the condition through the print and electronic media is commenced as this will go a long way in quelling the various misconceptions about the condition among the populace and also encourage early presentation and hence prevent complications that accompany surgical operation of huge fibroids. Qualified health care professionals should also be involved in the public enlightenment and campaign.

## Figures and Tables

**Table 1:** Sociodemographic characteristics of the respondents (*n* = 300).

Characteristics	Frequency (*N*)	Percentage (%)
Age		
20–24	3	1.0
25–29	10	3.3
30–34	84	28.0
35–39	88	29.3
40–44	50	16.7
45–49	65	21.7
**Age range = 20–49 years Mean age ± SD = 30.6 ± 1.34**		

Marital status		
Single	72	24.0
Married	200	66.7
Divorced	25	8.3
Widowed	3	1.0

Parity		
0	158	52.7
1	61	20.3
2	30	10.0
3	29	9.7
4	15	5.0
>5	7	2.3
Mean parity = 1.65 SD ± 1.97		

Occupational status		
Professional	40	13.3
Semiprofessional	72	24.0
Skilled	44	14.7
Unskilled	56	18.7
Unemployed	88	29.3

Total	300	100.0

**Table 2:** Clinical features in the respondents (*n* = 300).

Clinical features	Frequency (*N*)	Percentage (%)
Timing of presentation after onset of symptoms		
Early (<6 months)	40	13.3
Late (≥6 months)	260	86.7

Complaint at presentation		
Menorrhagia	223	74.3
Urinary frequency	30	10.0
Abdominal mass	264	88.0
Irregular bleeding	40	13.3
Inability to conceive	158	52.7

Uterine size at presentation		
<20-week size	34	11.3
20–25-week size	230	76.7
26–30-week size	30	10.0
>30-week size	6	2.0

Total	300	100.0

**Table 3:** Knowledge of fibroids.

Knowledge	Frequency (*N*)	Percentage (%)
Awareness of uterine fibroids (*n* = 300)		
Yes	296	98.6
No	4	3.4

Source(s) of information (*n* = 300)		
Parents and relations	82	27.3
Television	55	18.3
Radio	87	29.0
Newspaper	20	6.7
Health workers/hospital	56	18.7

Knowledge of the predisposing factors		
Obesity	48	6.0
Women who had never had a child	100	33.3
Black women	107	35.7
People with family history of fibroids	78	26.0
No knowledge	4	1.3

**Table 4:** Perception of respondents to fibroid.

Perception	Frequency (*N*)	Percentage (%)
Beliefs (*n* = 462)		
Fibroid is only seen in infertile women	97	32.3
Fibroid is a spiritual problem	207	67.0
Fibroid affects childbearing	158	52.7

Treatment seeking behaviours of respondents (*n* = 643)		
Fibroid is best treated with herbs	93	31.0
Fear of surgery due to its complications	250	83.3
Treatment best given in spiritual homes	201	67.0
Appeasing the goddess of water	10	3.3
Medical treatment with drugs	89	29.7

**Table 5:** Drugs used by respondents for treatment of fibroids.

Drugs used	Frequency (*N*)	Percentage (%)
No drugs	16	5.3
Clomiphene citrate	29	9.7
Bromocriptine	29	9.7
Cod liver oil	21	7.0
Tianshi	59	19.7
GNLD	34	11.3
Yetkem herbal drugs	40	13.3
Oko-Oloyun herbal drugs	72	24.0

Total	300	100.0

*Phytoestrogen containing compounds: Tianshi, GNLD, Yetkem herbal drugs, and Oko-Oloyun herbal drugs.
